# Human epithelial cell cultures from superficial limbal explants

**Published:** 2011-02-01

**Authors:** D. Ghoubay-Benallaoua, E. Basli, P. Goldschmidt, F. Pecha, C. Chaumeil, L. Laroche, V. Borderie

**Affiliations:** 1 Institut de la Vision, UPMC Univ Paris 06, UMR_S 968 / INSERM, U968 / CHNO des XV-XX / CNRS, UMR_7210, Paris, France; 2 Laboratoire du Centre Hospitalier National d’Ophtalmologie des Quinze-Vingts, Paris, France; 3 Banque de Tissus, Établissement Français du Sang, Paris, France

## Abstract

**Purpose:**

To study the kinetics of growth and the phenotype of cells cultured from human limbal explants in a cholera toxin-free medium with no feeder cell layer.

**Methods:**

Human organ-cultured corneas were used to prepare limbal explants (full-thickness and superficial limbal explants) and corneal stromal explants. Cell growth kinetics and phenotypes were assessed by cultivating explants in cholera toxin-free Green medium. Epithelial and progenitor cell markers were assessed by immunocytochemistry, flow cytometry, and Reverse Transcription and Polymerase Chain Reaction (RT-PCR).

**Results:**

The successful epithelial cell growth rates from full thickness limbal explant and superficial limbal explant tissues were 41 and 86%, respectively (p=0.0001). The mean cell area and the percentage of small cells in superficial and full-thickness explant cultures were, respectively, 317 µm^2^ and 429 µm^2^, and 8.9% and 1.7% (p<0.001). The percentage of positive cells in superficial and full-thickness limbal explant cultures as assessed by immunocytochemistry were the following: broad spectrum cytokeratins (cytokeratins 4, 5, 6, 8, 10, 13, and 18 [MNF116]), 82%/37% (p=0.01); cytokeratin 3 (CK3), 74%/25% (p=0.009); cytokeratin 19 (CK19), 46%/25% (p=0.19); vimentin, 56%/53% (p=0.48); delta N p63α, 54%/0% (p<0.001); and ABCG2, 5%/0% (p=0.1). Flow cytometry showed a higher percentage of small cells, a higher percentage of MNF116+ cells, and stronger expression of progenitor-associated markers in superficial than in full-thickness explant cultures. For superficial limbal explant cultures, analysis of the expression profiles for various mRNAs at the end of 21 days of culture showed high levels of expression of the mRNAs encoding *CK3*, vimentin, and *CK19*. The expression of mRNA of *delta N p63α* and *ABCG2* was weaker. Cultures obtained from full-thickness limbal explants featured no expression of mRNA of *CK19*, *delta N p63α*, and *ABCG2*, whereas mRNAs encoding *CK3* and vimentin were detected. Human corneal stromal explants cultured with the same medium featured late cell growth, large mean cell area (2,529 µm^2^), no expression of cytokeratins, delta N p63α, and ABCG2, and high expression of vimentin.

**Conclusions:**

Superficial limbal explants appear to be superior to full-thickness limbal explants for growing human limbal epithelial cells. Preparation of explants using surgical facilities (i.e., operating microscope and microsurgical blades) led to a dramatic increase in the percentage of successful cultures, higher epithelial cell growth, decreased fibroblast contamination, and better preservation of limbal epithelial progenitors.

## Introduction

In recent years, understanding of the limbal cell progenitor has led to a change in the management of ocular surface disorders. Kenyon and Tseng [1] first reported transplantation in 1989 with functional and anatomic results in a series of 21 limbal autografts. Further reports confirmed the efficacy of this approach then widened to include allografts obtained from cadaveric or living related donors [2-7].

Several issues have been hypothesized to explain the corneal epithelium renewal [8]. Among these theories, the limbal epithelial progenitors are currently thought to be the source of corneal epithelial cells. The cultured limbal epithelial cells are able to restore the corneal surface in patients with complete limbal deficiency [9]. The limbal epithelial basal layer contains cells with a phenotype suggesting undifferentiated stem-like cells, such as expression of the alpha isoform of Delta N p63 [10-12]. When cultured ex vivo the limbal epithelial cells seem to have the ability to express different phenotypes since they produce nestin which is a marker of neural stem cells [13-15]. The long-term self-renewal of limbal basal epithelial cells in addition to their plasticity and ability to give rise to mature cells are strong arguments in favor of adult corneal pluripotent cells in the limbal epithelium. Nevertheless, in a mouse model it was shown that the limbus does not contribute to steady-state corneal renewal but to corneal repair [16].

The majority of clinical studies were performed with explants originated from fresh limbal tissue. However, James et al. [17] compared epithelial cultures from fresh and preserved tissue and reported a lower potential for preserved material, with heterogeneous success rates. Limbal explants obtained from 3- to 4-weeks organ-cultured human corneas supported expansion of poorly differentiated epithelial cells when maintained in culture during 3 weeks [18].

The initial Green medium consists of Dulbecco’s Modified Eagle’s Medium and Ham F12 medium with fetal bovine serum, cholera toxin, insulin, hydrocortisone, L-glutamine, and antibiotics [19]. It has been further modified with addition of adenine, tri-iodo-thyronin, HEPES buffer, and amphotericin B [13,17]. An extensive screening of all these components for their production process and origin showed an optimum level of safety (i.e., chemical products or products of controlled and documented animal origin) for all components except cholera toxin which is obtained after bacterial cultures on bovine brain broth and fetal calf serum. To minimise the risks of transmission of conventional (bacteria, fungi and viruses) and non conventional (prions) infectious agents to human, fulfilling the requirements of the French Health Authority and the European Union directives, the present work was performed culturing cells in a cholera-toxin free media and using sterile irradiated fetal calf serum obtained from New Zealand, where it was established that bovine spongiform encephalitis was absent. In fact bovine brain and fetal calf serum are used for producing cholera toxin and no information indicates that these bovine products are obtained in countries free from bovine spongiform encephalitis. Our medium has been approved by the French National Regulation Agency (AFSSaPS) for clinical use in humans in a prospective clinical trial.

The objective of this work was to study the kinetics of growth and the phenotype of cells cultured from human superficial limbal explants with the goal of minimizing contamination with fibroblast and to preserve limbal niche during culture in the medium approved by the French National Regulation Agency (cholera toxin-free medium) with no feeder cell layer. The expression of epithelial and progenitor cell associated markers CK3, CK19, p63, ABCG2, and vimentin was assessed.

## Methods

This study was performed according to the tenets of the Declaration of Helsinki and it followed international ethic requirements for human tissues.

### Donor corneal tissue

Human donor corneas discarded before transplantation due to low endothelial cell counts (eye bank corneas) and corneoscleral rims obtained during surgery after 8-mm trephination of the graft (surgical corneas) were used. Corneoscleral rims from the eye bank corneas were obtained using an 8-mm trephine.

### Preparation of explants

Full-thickness limbal explants were prepared under a laminar flow. Descemet’s membrane was removed using a scalpel and full-thickness limbal rims were isolated by removing the sclera with scissors. Superficial limbal explants were processed in the operating room under an operating microscope. A stromal dissection between the anterior and the mid stroma was performed using a 15° blade and the sclera was carefully removed with scissors resulting in superficial limbal rims. For the growth assay a 2.5-mm trephine was used to obtain 9 round explants with homogeneous areas of limbal epithelium. For all other experiments 6 explants with homogeneous length (4 mm) were obtained from each limbal rim using scissors. Corneal stromal explants were prepared using the central cornea after 8-mm trephination of eye-bank corneas. The epithelium and Descemet’s membrane were first removed and 2-mm stromal explants were prepared with a scalpel.

### Culture media

Limbal explants were cultured in cholera toxin-free Green medium. The medium was composed of a 3:1 mixture of calcium-free Dulbecco’s Modified Eagle’s Medium (Dutscher, Brumath, France) and Ham F12 medium (Invitrogen, Cergy Pontoise, France) with 10% fetal bovine serum (Invitrogen), 1 mM/ml HEPES buffer (Invitrogen), 5 µg/ml human recombinant insulin (Actrapid^®^; Novo Nordisk, Paris, France), 0.4 µg/ml hydrocortisone (Pharmacia, Pfizer, Paris, France), 4 µM/ml L-glutamine (Invitrogen), 2 pM/ml tri-iodo thyronine (Sigma, Saint Quentin en Yvelines, France), 200 nM/ml adenine (Sigma), 100 IU/ml penicillin (Invitrogen), 100 µg/ml streptomycin (Invitrogen), 0.25 µg/ml amphotericin B (Invitrogen), and 10 ng/ml human recombinant Epithelial Growth Factor (EGF; Sigma).

### Cell culture

In a first series of experiments, the full-thickness limbal explants or the superficial limbal explants either sutured on a plastic lamella (320 mm^2^; one explant per lamella; Thermanox, Nunc, Illkirch, France) epithelial side up were cultured using 6- (907 mm^2^) or 24-well (201 mm^2^) plates (Becton Dickinson, Rungis, France) with 2 ml of medium. To determine whether cultured cells corresponded to epithelial cells, stromal fibroblasts, or mixed cultures, a second series of experiments was performed. Full-thickness and superficial limbal explants (epithelium either side up or down) and stromal explants obtained from two corneas from the same donor were cultured using 6-well plates. Finally, single cell suspensions isolated from limbal tissue were cultured. Briefly, the whole limbal rim was incubated with 1.2 Units/ml dispase II at 37 °C for 1 h. The epithelial sheets were then collected and treated with 0.125% trypsin at 37 °C for 15 min to isolate single cells. No feeders were used to grow cells. The medium was changed three times a week and cells were cultured for three weeks at 37 °C with 5% CO_2_.

### Morphological analysis

Morphological analysis was performed twice a week with a phase contrast light microscope. Peripheral explant cell sheet areas were calculated after reconstitution of images using the Image J 1.34 software (National Institutes of Health, Bethesda, MD) after 1, 2, and 3 weeks of culture. Confocal microscopy was performed on 3-week cultures using the Heidelberg Retina Tomograph III with the Rostock Cornea Module (HRT III/RCM; Heidelberg Engineering GmbH, Heidelberg, Germany).

### Morphometric analysis

After three weeks cells were fixed with 4% paraformaldehyde solution for 10 min at room temperature. In each well, at least 300 cells from 3 fields were analyzed and 36 images (3 per well) were acquired with a color camera for computerized analysis. For each image, The Image J software allowed to study morphology after individual contours were designed manually. The mean values for cell area, Feret diameter, and percentage of small cells (less than 16 µm^2^) were assessed for each well.

### Growth assay

Limbal explants trephined at 2.5 mm were cultivated in 24-well plates. After 7, 14, and 21 days, cells were dissociated enzymatically and counted. The equation giving the number of cells in culture (n_t_) at t time is the following: n_t_=n_0_ × e^(t/T)xln2^, where n_0_ is the number of cells in the explant at the beginning of culture and T is the time period of the cell cycle. The equation which best-fitted the observed curve of growth was determined using Excel software (Microsoft Excel; Microsoft Corporation, Paris, France).

### Immunocytochemistry

Immunocytochemical staining was performed to evaluate the expression of different molecular markers and to identify limbal epithelial cells and progenitors. Six slides were immunostained for each marker for superficial limbal explants and six for full-thickness limbal explants cultured cells. The technique was developed with the objective to get no background staining which allows easy differentiation of stained and unstained cells and easy counting of stained cells. Normal corneas obtained after enucleation for choroidal melanoma were used as control tissues. Tissues were fixed in paraffin and stored at −20 °C. Frozen sections cut with a Microtom (Leica RM2145; Leica, Paris, France) were collected on slides (Superfrost; ThermoScientific, Illkirch, France) and were put in histosol and rehydrated in 100%, 90%, and 70% ethanol and double distilled water. Cultured cells were enzymatically dissociated and concentrated by cytospin. After washing in PBS, the tissue sections and cultivated cells were fixed for 10 min with 4% paraformaldehyde and incubated for 30 min in PBS containing 1% BSA (BSA) and 0.3% Triton X 100 to permeabilize the cells and to block non-specific staining. The endogenous peroxidases were quenched with 0.3% H_2_O_2_ during 10 min. Cells were incubated for 30 min at room temperature with primary antibodies against cytokeratin 3 (1:200; Clone AE-5; Dako, Trappes, France), cytokeratins 4, 5, 6, 8, 10, 13, and 18 (1:100; MNF116; Dako), cytokeratin 19 (1:50; clone BA17, Dako), vimentin (1:200; clone V9, Dako), Delta N p63α (1:50; clone 4A4, Dako) and ATP-binding cassette, subfamily G, member 2 (ABCG2; 1:20; clone CDw338, BD PharMingen, San Diego, CA) followed by incubation with the biotinylated secondary antibody using a LSAB2 system-HRP Kit (Dako) according to the manufacturer’s instructions. DAB was used as peroxidase substrate and specimens were counterstained with hematoxylin.

### Flow cytometry analysis

The flow cytometry analysis was performed on a BD FACSCalibur system (Becton Dickinson). The staining of each population, combined with the forward scatter channel (FSC) and the side scatter channel (SSC) data, identifies cells present in a sample and it allows counting the relative proportions of each. Cell suspensions (10^5^cells/ml) in PBS/BSA buffer were fixed with 4% paraformaldehyde (Fix Buffer I; BD Bioscience) for 10 min at 37 °C. After washing with PBS/BSA, cells were permeabilized with Perm Buffer III (BD Bioscience) and incubated for 30 min at 4 °C. Aliquots were distributed into different test tubes for primary antibody binding (CK3, vimentin, CK 4, 5, 6, 8, 10, 13, and 18, CK19, DeltaN p63α, and ABCG2) and incubated at room temperature for 30 min. An anti-mouse IgG1 FITC or IgG2a FITC secondary antibody was added, and cells were incubated for 30 min at room temperature. Relative size, granularity or internal complexity, and relative fluorescence intensity were registered with the Cell Quest Pro software (BD Bioscience).

### Assessment of specific messengers RNAs in cell culture by reverse transcription and Polymerase Chain Reaction (RT–PCR)

Total RNA was isolated using the MagNA Pure Compact RNA isolation Kit (Roche Diagnostics, Mannheim, Germany) after 21 days of culture according to the manufacturer’s instructions. The RNAs were quantified by measuring absorption at 260 nm. RT–PCR was performed using the Qiagen One Step RT–PCR Kit (Qiagen, Courtaboeuf, France) which provides enzymes for both the reverse transcription and the PCR. The first-strand cDNA was synthesized after incubation at 50 °C for 30 min and PCR conditions were 95 °C for 15 min followed by 35 cycles of 94 °C for 30 s, 60 °C for 30 s, 72 °C for 1 min, and finally at 72 °C for 10 min. The sequences of the primers for human Cytokeratins 3 and 19, vimentin, Delta N p63α, *ABCG2*, and β-actin (*ACTB*), a housekeeping gene used as an internal control, are presented in Table 1. The amplified products were separated using 2% agarose gel electrophoresis stained with ethidium bromide.

**Table 1:** Primers selected for PCR testing after reverse transcription.

**Gene name**	**Sequence**	**PCR product size (bp)**
Cytokeratin 3	F: 5′-CGTGGTCAGCAGCAGCACGA-3′	258
	R: 5′-CGGTTGCTGGCCGAGCTGAA-3′	
Vimentin	F: 5′-TGGCCGACGCCATCAACACC-3′	257
	R: 5′-CACCTCGACGCGGGCTTTGT-3′	
Cytokeratin 19	F: 5′-GGTTGCTCCGTCCGTGCTCC-3′	270
	R: 5′-TTCTCGTTGCCCGCCAGCAG-3′	
ABCG2	F: 5′-AGTTCCATGGCACTGGCCATA-3′	395
	R: 5′-TCAGGTAGGCAATTGTGAGG-3′	
DeltaN p63α	F: 5′-TGGCAAAATCCTGGAGCCAGAAGA-3′	104
	R: 5′-GTGGCTCACTAAATTGAGTCTGGGC-3′	
β-actin	F: 5′-TCATGTTTGAGACCTTCAACACCC-3′	602
	R: 5′-GTACTTGCGCTCAGGAGGAG-3′	

### Statistical analysis

Non-parametric tests (χ^2^, Spearman rank correlation coefficient, Kruskal–Wallis ANOVA, and Mann–Whitney test) were used for statistical analysis with the Statistica 6.1 software (StatSoft Inc., Maisons-Alfort, France); a p<0.05 was considered statistically significant.

## Results

### Donor tissue

Thirty-two human corneas were used in this study. The average donor age was 66±16 years (SD, range 32 to 80 years). Time from death to tissue procurement ranged between 9 and 47 h (mean 23±1 h). All corneas had been organ-cultured as previously described [20,21] for an average of 18±5 days before trephination.

### Comparison of explant preparation techniques

Full-thickness limbal explants were first used for growing limbal epithelial cells. Cell growth appeared to be closely linked to adhesion of explants to the bottom of the culture well. Then preparation of superficial explants and fixation of explants was developed to improve adhesion.

**Figure 1:**
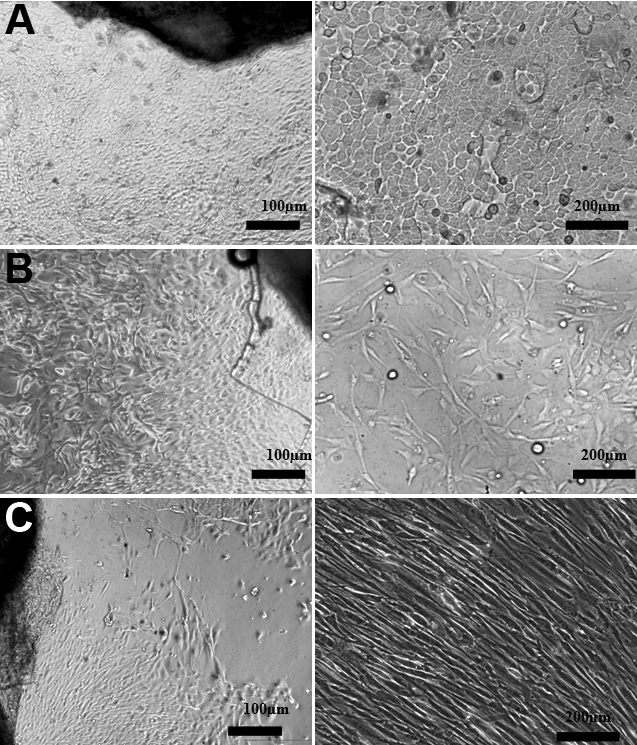
Limbal epithelial cells cultured from human explants. **A**: Superficial limbal explant with polygonal cells covering the well after three weeks. **B**: Polygonal and fibroblast-like cells from full-thickness limbal explant. **C**: Fibroblast-like cells from stromal explant.

In successful cultures, after three weeks, the limbal epithelial cells covered the well surface. Only polygonal cells were observed when superficial limbal explants were cultivated (Figure 1A). Conversely, two types of cells (polygonal cells and fibroblast-like cells) were observed for full-thickness limbal explants (Figure 1B) and one type of cells, spindle-shaped, large and flat, were observed for stromal explant culture (Figure 1C).

In the first series of experiments, significant differences in the percentage of confluent cultures after 3 weeks were found according to explant preparation technique (Table 2). This figure was significantly higher for superficial explants (p=0.0001) and for superficial explants sutured on plastic lamella (p=0.00001) compared to full-thickness explants. However no significant differences (p=0.65) were found between non-sutured superficial explants and superficial explants sutured on plastic lamella.

**Table 2:** Confluent cultures (%) after three weeks according to the explant preparation technique.

**Three-week cultureb(cultured using 6-well plates)**	**Full-thickness limbal explant (66 explants; explant length: 4 mm)**	**Superficial limbal explant (33 explants; explant length: 4 mm)**	**Superficial limbal explant sutured on plastic lamella (22 explants; explant length: 4 mm)**	**p (χ^2^ test)**
Confluent sheet	27 (41%)	27 (82%)	19 (86%)	0.00001
Non confluent sheet	39 (59%)	6 (18%)	3 (14%)	

**Figure 2:**
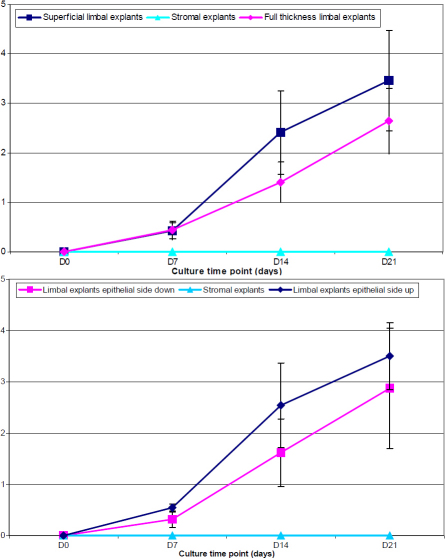
Graphic representation of the evolution of the cell sheet area of human limbal explants and stromal explants.

In the second series of experiments (Figure 2), no cell growth was observed from stromal explants during the first 3 weeks of culture. Stromal fibroblast growth started after 40 days of culture. Dissociated limbal epithelial cells did not attach to the bottom of the well which resulted in no cell growth. The cell sheet area was significantly larger after 14 (p=0.007) and 21 days (p=0.02) of culture and the number of cells per well after 21 days of culture was significantly higher (p=0.002) with superficial than with full-thickness limbal explants (Figure 2, Table 3). The cell sheet area was significantly larger after 7 (p=0.001) and 14 days (p=0.03) of culture, but not after 21 days (p=0.26), with limbal explants epithelial side up as compared with limbal explants epithelial side down (Figure 2). No significant differences in the number of cells per well after 21 days of culture were found between limbal explants epithelial side up and limbal explants epithelial side down (p=0.59; Table 3).

**Table 3:** Number of cells per well after three weeks according to the explant preparation technique.

**Explant**	**Number of cells per well after 21 days of culture**
Limbal explants epithelial side up	242,000±160,008
Limbal explants epithelial side down	149,667±99,440
Superficial limbal explants	299,500±107,785
Full thickness limbal explants	92,167±56,538
Stromal explants	0

Morphometry results are presented in Table 4. Cells showed higher average cell area, average cell perimeter, and average Feret diameter when full thickness limbal explants were cultured as compared with cultures obtained from superficial limbal explants (p<0.001). Fibroblasts cultured from stromal explants featured dramatically larger cell area, perimeter, and diameter (p<0.001).

**Table 4:** Morphometry of human cells cultured from limbal and stromal explants.

**Explant preparation**	**Cell area (µm^2^)**	**Percentage of small cells (cell area <16 µm^2^)**	**Cell perimeter (µm)**	**Feret diameter (µm)**
Superficial limbal explants	317±116	8.9%	75±17	28±7
Full-thickness limbal explants	429±108	1.7%	79±10	31±3
Stromal explants	2,529±1,098	0.0%	488±163	231±79

### Growth assay

**Figure 3:**
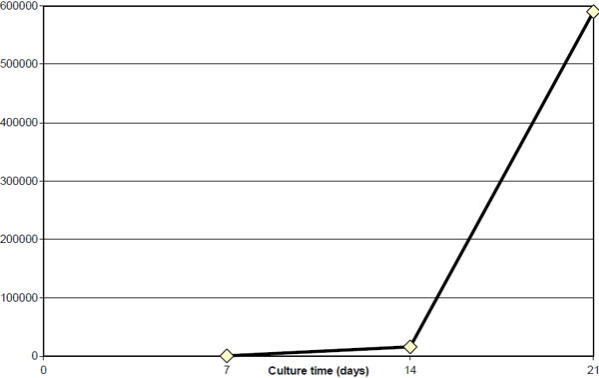
Graphic representation of the curve growth of cultured human limbal epithelial cells. Using the equation which best-fitted the observed curve of growth the resulting average time period of the cell cycle was 1 day.

From a superficial limbal explant corresponding to 1/6 of the limbal circumference, an average of 5.9×10^5^ cells could be obtained after 21 days of culture (Figure 3). The number of cells obtained after 21 days did not significantly correlate with donor age (p=0.66), death-to-preservation time (p=0.71), and organ culture time (p=0.58). The average cell sheet area was 5 mm^2^ after 7 days, 140 mm^2^ after 14 days, and 227 mm^2^ after 21 days.

### Confocal microscopy

**Figure 4:**
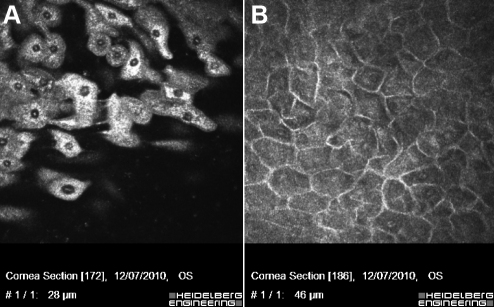
Confocal microscopy of 3-week cultures of superficial limbal explants. **A**: Large flattened superficial cells. **B**: Small basal cells.

Confocal microscopy of 3-week cultures showed a multilayered epithelial cell sheet, with small basal cells and large flattened superficial cells (Figure 4).

### Immunocytochemistry

**Figure 5:**
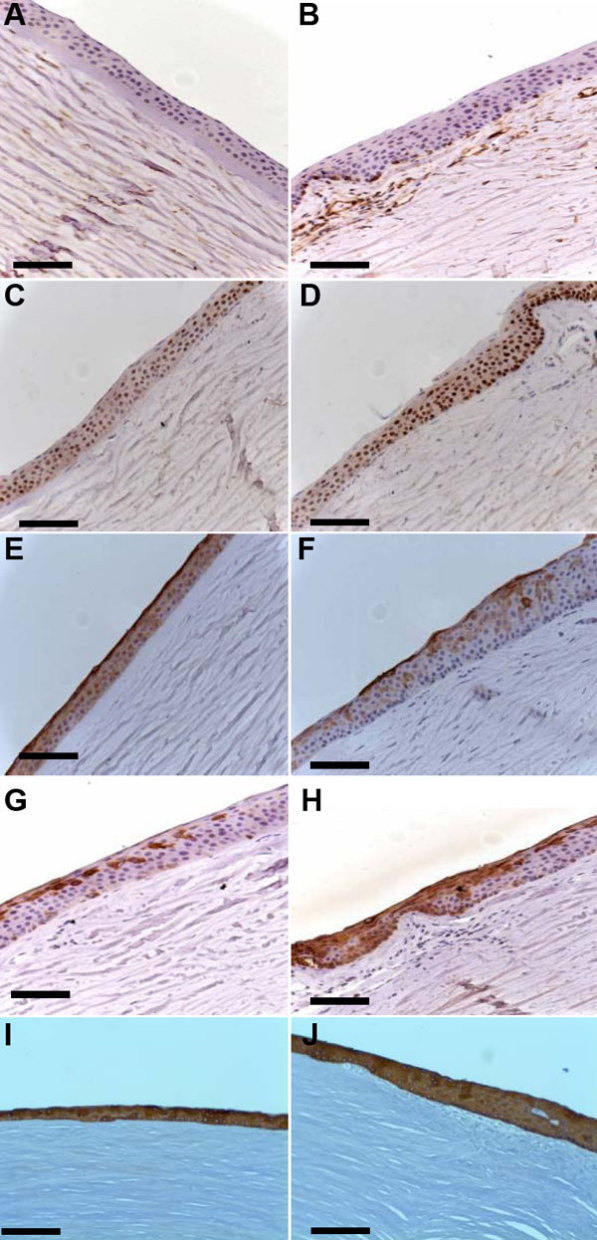
Immunohistochemical staining of  normal central cornea and limbus. Vimentin (**A**, **B**), delta N p63α (**C**, **D**), cytokeratin 3 (**E**, **F**), cytokeratin 19 (**G**, **H**), and broad spectrum cytokeratins (MNF116; **I**, **J**) in normal central cornea and limbus. Vimentin is not detected in corneal epithelial cells (**A**) but is detected in the basal limbal epithelial layer and stromal cells (**B**). Delta N p63α is strongly expressed in the basal layer of the limbus (**D**). The entire superficial limbal epithelial layer and cells in the mid layer are positive for CK3 (**F**). CK19 labels the basal and superficial limbal epithelial layers whereas cells in the mid layer are not stained by anti-CK19. Bars: 200 µm; magnification: 10×.

**Figure 6:**
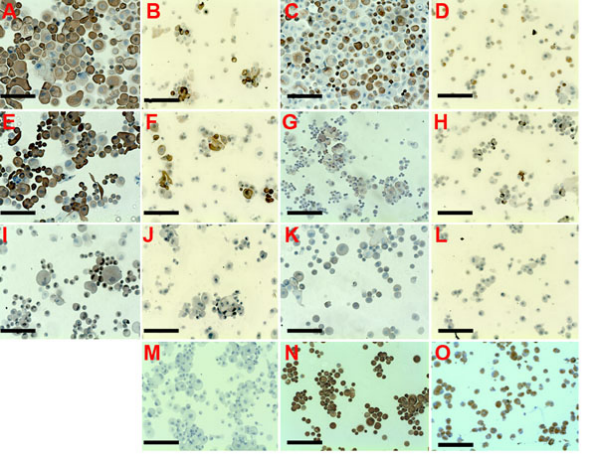
Epithelial cells obtained from full thickness and superficial limbal explants cultured for 3 weeks in cholera toxin-free Green medium. Staining for CK3 (**A**: superficial limbal explants; **B**: full thickness limbal explants), vimentin (**C**: superficial limbal explants. **D**: full thickness limbal explants), broad spectrum cytokeratins (CK4, 5, 6, 8, 10, 13, and 18; **E**: superficial limbal explants; **F**: full thickness limbal explants), CK19 (**G**: superficial limbal explants; **H**: full thickness limbal explants), delta N p63α (**I**: superficial limbal explants; **J**: full thickness limbal explants), ABCG2 (**K**: superficial limbal explants; **L**: full thickness limbal explants) of limbal cells; **M**: negative control; **N**: positive control (human corneal epithelial cells stained with CK3). **O**: keratocytes stained with vimentin. Bars: 200 µm; magnification: 10×.

Figure 5 shows that vimentin, cytokeratin 3, cytokeratin 19, and delta N p63α are expressed in normal human corneas (limbus and central cornea). MNF116 antibody (recognizes a group of 7 cytokeratins characteristic of epithelial cells) was expressed in all corneal and all limbal epithelial cells (Figure 5I and Figure 5J). The broad spectrum cytokeratins was not expressed by stromal cells. When limbal epithelial cell from explants were cultured for 3 weeks, the MNF116 antibody stained 82±19% of cells obtained from superficial limbal (Figure 6E) and 37±3% of cells obtained from full thickness limbal explants (Figure 6F; p=0.01). Cytokeratin 3, a corneal specific marker, was strongly expressed by all corneal epithelial cells (Figure 5E) and by the superficial limbal epithelial cells (Figure 5F). For superficial limbal explants 74±19% were CK3 positive (Figure 6A) and only 25±6% for full thickness limbal explants (Figure 6B; p=0.009). Vimentin was detected in 100% of limbal basal epithelial and stromal cells (Figure 5A) but was undetectable in the corneal epithelial layers (Figure 5B). Vimentin was expressed in 56±18% and 53±5% of cells from superficial (Figure 6C) and full thickness (Figure 6D) limbal explants respectively (p=0.48). Delta N p63α protein was detected in the nucleus of limbal epithelial basal cells (Figure 5D). When superficial limbal explants were cultured, delta N p63α was detected in 54±20% (Figure 6I) but undetectable (0%) in cells cultured from full thickness limbal explants (p<0.001). Cytokeratin 19 (a proposed marker for skin hair follicle progenitor stem cell) was highly expressed in the cytoplasm of basal and superficial limbal epithelial cells (Figure 5H) and it was expressed by 46±27% cells when superficial limbal explants were cultured (Figure 6G) and 25±12% when it was full thickness limbal explants (Figure 6H; p=0.19). ABCG2 has been proposed as one of the universal markers for progenitor cells. However, under our experimental conditions the number of cells positive for ABCG2 in cells cultured from full thickness limbal explants (Figure 6L) was negative, while those obtained with superficial limbal explants culture was 5±7% (Figure 6K; p=0.1). In the positive control all the human corneal epithelial cells expressed CK3 (Figure 6N) but not vimentin. Vimentin was expressed by all cells cultured from stromal explants (Figure 6O). CK3 and broad spectrum cytokeratins (MNF116) were not expressed by these cells.

In the second series of experiments, 100% of cells cultured from stromal explants expressed vimentin but not cytokeratins. Differences in the percentage of cells stained by AE5, MNF116, and vimentin antibodies between superficial and full-thickness limbal explants and between limbal explants epithelium side up and limbal explants epithelium side down did not reach statistical significance (p>0.05; Table 5). When superficial limbal explants were cultured, delta N p63α was detected in 50% but undetectable (0%) in cells cultured from full thickness limbal explants.

**Table 5:** Expression of CK3, CK4, 5, 6, 8, 10, 13, 18, vimentin, delta N p63α, CK19, and ABCG2 in primary cultured human epithelial cells assessed by immunocytochemistry.

**Explant**	**CK4,5,6,8,10,13,18**	**CK3**	**Vimentin**	**Delta N p63α**	**CK19**	**ABCG2**
Limbal explants epithelium side up	90±13%	94±6%	28±14%	28±5%	37± 29%	0%
Limbal explants epithelium side down	95±6%	86±13%	37±9%	22±17%	22±19%	0%
Superficial limbal explants	97±3%	95±3%	32±7%	50±18%	42±31%	0%
Full thickness limbal explants	88±12%	85±13%	33±17%	0%	17±5%	0%
Stromal explants	0%	0%	100%	0%	0%	0%

### Flow Cytometry

**Figure 7:**
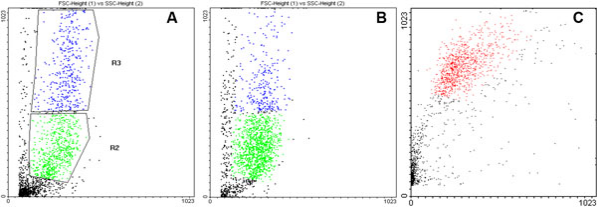
Flow cytometry analysis of cultured cells. Single cell suspensions were analyzed according to their size (FSC) and granularity (SSC) profiles. A representative dot-plot was shown for full thickness (**A**), superficial limbal explants (**B**), and stromal explants (**C**). R2: cells with a small size; R3: cells with a large size.

Figure 7 shows the dot plots corresponding to the analysis of cell suspensions from limbal explants cultured for three weeks by flow cytometry. The proportion of cells with a small size (polygonal cells) was higher in cultures obtained with superficial (83%) than with full thickness limbal explants (68%) and stromal explants (10%). Ninety percent of cells were large when stromal explants were cultured. Thirty-two percents and 17% of cells were large when full thickness limbal explants and superficial limbal explants were cultured, respectively.

High levels of expression of CK3, CK4, 5, 6, 8, 10, 13, and 18 was observed in superficial and full thickness limbal explants cultured cells. Expression of vimentin was higher in full thickness than in superficial limbal explant cultured cells. Lower levels of expression were found for delta N p63α and ABCG2 in superficial limbal explants cultured cells and no expression was found in full thickness limbal explant cultured cells (Table 6).

**Table 6:** Expression of CK3, CK4, 5, 6, 8, 10, 13, 18, vimentin, delta N p63α, ABCG2 and CK19 in primary cultured human epithelial cells assessed by flow cytometry.

**Markers**	**CK3**	**CK4,5,6,8,10,13,18**	**Vimentin**	**CK19**	**Delta N p63α**	**ABCG2**
Superficial limbal explants	67%	72%	63%	48%	7%	4%
Full-thickness limbal explants	66%	58%	80%	6%	0%	0%
Stromal explants	0%	0%	70%	0%	0%	0%

### RT- PCR

**Figure 8:**
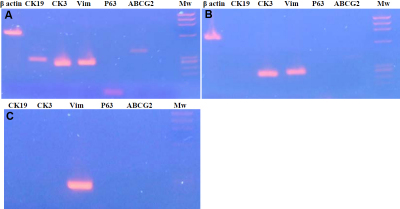
Electrophoresis of amplicons obtained after reverse transcription of mRNAs extracted from cultured cells. Primers used for PCR bracket selectively the complementary sequences of *CK3*, *CK1*9, vimentin, *ABCG2*, and *delta N p63α*. RNAs were extracted after 3 weeks of cultures of **A**: superficial limbal explants, **B**: full-thickness limbal explants, and **C**: stromal explants.

For superficial limbal explant cultures, the analysis of the expression profiles for various RNAs (*CK3*, *CK19*, vimentin, *delta N p63α*, and *ABCG2*) at the end of 21 days of culture showed high levels of expression of the mRNAs encoding *CK3*, vimentin, and *CK19*. The expression of mRNA of *delta N p63α* and *ABCG2* was weaker (Figure 8). Cultures obtained from full-thickness limbal explants featured no expression of mRNA of *CK19*, *delta N p63α*, and *ABCG2*, whereas mRNAs encoding *CK3* and vimentin were detected. Fibroblasts cultured from corneal stromal explants featured no expression of mRNA of *CK19*, *CK3*, *delta N p63α*, and *ABCG2*, whereas mRNA encoding vimentin was detected.

## Discussion

The growth potential of limbal epithelial cells expanded from full thickness and superficial limbal explants was studied. The successful epithelial cell growth rates from these tissues were 41 and 86% in full-thickness and superficial limbal explants cultures, respectively. The time to reach confluence was similar in both groups (14–21 days). Using our culture conditions which do not include feeder cells, no cell growth could be obtained from corneal stromal explants nor from dissociated limbal epithelial cells during the first 3 weeks of culture. Conversely, cell growth could be obtained from superficial and full-thickness limbal explants, either epithelial side up or down, with the limbal explants epithelial side up showing higher cell growth during the first 2 weeks.

Although both culture systems produce limbal epithelial sheets, the superficial limbal explants promoted more rapid expansion of the epithelial cell population than the full-thickness limbal explants culture. The third culture system (stromal explant) produces cells after 40 days of culture. The cells in superficial explants cultures appeared to be smaller and more uniform than in full-thickness explants. The cells in stromal explant cultures were large and flat and they were aligned in parallel clusters. The proportion of small-size cells was higher in superficial than in full-thickness limbal explant culture. These cells were absent in stromal explant culture. Conversely, superficial limbal explant allowed growth of epithelial cells with fewer large cells.

In this study, the phenotype of primary cultured cells was assessed by immunostaining with antibodies for progenitor markers such as delta N p63α, CK19, and ABCG2, and differentiation markers such as MNF116, CK3, and vimentin. Quantitative analysis of cell phenotype was performed which has not often been reported by previous studies.

The immunostaining using anti CK3 labels, a terminal differentiation indicator of the corneal epithelium, allowed identification of differentiated cells on both the corneal and limbal epithelium. CK3 staining was more important in superficial than in full-thickness limbal explants culture and it was absent in stromal explant culture.

MNF116 was the alternative name of 7 cytokeratins which are characteristic of epithelial and trichocytic cells and cytokeratins 4, 5, 6, and 8 members of the type I neutral to basic subfamily and cytokeratin 10, 13, and 18 members of type I acidic subfamily [22]. The expression of this cytokeratins was higher in superficial limbal explants culture than in full-thickness one.

Vimentin is not expressed in situ by limbal and corneal epithelial cells. However, the present study demonstrates that cultured limbal epithelial cells obtained from the two preparations (superficial and full-thickness limbal explants) express vimentin, probably by cell to matrix interactions or the presence of fibroblasts in both culture techniques. At least a low percentage of vimentin-positive cultured limbal epithelial cells may correspond to limbal progenitors as suggested by strong expression of this marker in limbal basal epithelial cells. In stromal explants culture all cells express vimentin.

As a member of the cytokeratin family of intermediate filaments, CK19 has been suggested as a marker for the epidermal progenitors in skin follicles. In this study, we observed that CK19 labeling was found in the basal and superficial limbal epithelial layers whereas cells in the mid layer were not stained. Higher expression of CK19 was found in superficial than in full-thickness limbal explant cultures and no expression of this cytokeratin in stromal explant culture. Lauweryns et al. [23] identified a subpopulation of transitional cells in normal limbal tissue, that co-expressed CK19 and vimentin and suggested that they might be the progenitors.

The nuclear protein delta N p63α (member of the p53 family), was proposed as a marker to identify keratinocyte progenitors including limbal stem cells [10,24]. Here, the nuclear delta N p63α was expressed only in the basal layer of the limbal epithelium with no expression in the basal cells of the central corneal epithelium (transient amplifying cells). Nuclear delta N p63α was expressed in superficial limbal explant cell culture, evidenced by immunostaining, RT–PCR, and flow cytometry analysis. Conversely, it was not detected by any of these three methods in full thickness limbal explant culture. This shows that the superficial explant technique better preserves progenitors than the full-thickness explant technique. The percentage of delta N p63α positive cells in superficial explant culture was higher when assessed by immunocytochemistry than by flow cytometry. On the one hand, it could be hypothesized that cell preparation during immunocytochemistry resulted in higher permeabilization of the nuclear membrane. On the other hand, immunocytochemistry may overestimate the expression of delta N p63α as suggested by lower expression of the corresponding mRNA in RT–PCR. ABCG2 (member of the ATP-binding cassette) was detected in the cell membrane and cytoplasm of a few limbal basal epithelial cells, but not in the limbal suprabasal or corneal epithelial cells [25]. Cultures obtained from superficial limbal explants featured low expression of the ABCG2 protein and presence of its mRNA, whereas ABCG2 was not detected in full-thickness limbal explant cultures. Progenitor cells represent between 0.01% and 12% of the cell population [26] and in the limbus 10% of the basal cells are thought to act as progenitors [27]. Good agreement between immunocytochemistry, flow cytometry, and RT–PCR was found in this study for both preparation techniques.

Limbal epithelial cell grafts for clinical use can either be produced from intact limbal explants or from dissociated limbal epithelium [28] cultured with murine inactivated 3T3 fibroblasts. The former may have the advantage not to damage the limbal stem cells through enzymatic cell dissociation and to preserve the limbal niche during culture [29]. The latter may be associated with a lower risk of fibroblast growth during culture. Kim et al. showed that the immunostaining pattern of limbal epithelial cells from single cell cultures was similar to that of the explant cultures, with small cells strongly stained for p63 (20.9%) and CK19 (25%), while larger cells stained strongly with differentiation markers, CK3 (54%), involucrin (52.3%) and connexin 43 (58%) [28]. In our culture conditions, which do not include feeders, dissociated limbal epithelial cells did not adhere to the bottom of the well and no cell growth was observed.

In the present study, cultures obtained from superficial limbal explants consisted of more than 80% of epithelial cells as shown by broad spectrum cytokeratin expression. Most of these epithelial cells were differentiated corneal epithelial cells expressing CK3. Expression of delta N p63α, ABCG2, and CK19 demonstrates at least presence of progenitors. It is less easy to determine the origin of CK negative cells in culture (18% in average). The use of explants may lead to fibroblast growth in culture. However morphology of cells was clearly polygonal. Dramatic differences in cell morphometry, cell phenotype, and RNA expression were found between cultures of corneal stromal fibroblasts and cultures of limbal explants. In addition, fibroblast growth started after 40 days of culture. It is then unlikely that cultures of limbal explants contained a mixture of corneal fibroblasts and epithelial cells. Based on broad spectrum cytokeratin expression, the percentage of fibroblast contamination in cultures obtained from superficial limbal explants was 18% or less in the first series of experiments and 3% or less in the second one.

Culturing limbal explants epithelial side down did not improve the cell cultures. Cell growth was slightly slower during the first two weeks of culture with explants epithelial side down. However, after 3 weeks of culture, no significant differences in the cell sheet area, cell growth, and cell phenotype were observed between limbal explants epithelial side up and limbal explants epithelial side down. If the culture time had to be limited to 2 weeks, limbal explants should be cultured epithelial side up.

Corneoscleral rims obtained during surgery after graft trephination are easily available for culture and high number of rims can be used to grow high numbers of epithelial cells. The next step will consist in cell selection to obtain limbal stem cell-rich cell sheets. However, as no specific markers of limbal stem cells are currently available, strategies have to be developed to discard fibroblasts and differentiated epithelial cells. This approach should be of interest for patients requiring transplantation of allogenic cultured limbal stem cells (i.e., patients with no healthy contralateral eye) because the current available therapeutic approaches have shown a limited potential for restoring limbal function in the long-term (less than 70%) [30-33].

Cultures of superficial limbal explants from human corneas using a defined medium that strictly fulfils the current legal requirements for human grafts, allows obtaining in 3 weeks and in more than 80% of the explants, both corneal epithelial cells and progenitors.

In conclusion, superficial limbal explants appear to be the most suitable tissue for limbal epithelial cell growth. These findings were double checked by morphological analysis and immunostaining. Preparation of explants using surgical facilities (i.e., operating microscope and microsurgical blades) led to a dramatic increase in the percentage of successful cultures, higher epithelial cell growth, decreased fibroblast contamination, and better preservation of limbal epithelial progenitors.
